# Primary processing neuropils associated with the malleoli of camel spiders (Arachnida, Solifugae): a re-evaluation of axonal pathways

**DOI:** 10.1186/s40851-019-0137-z

**Published:** 2019-08-02

**Authors:** Andy Sombke, Anja E. Klann, Elisabeth Lipke, Harald Wolf

**Affiliations:** 1 University of Vienna, Department of Integrative Zoology, Althanstrasse 14, 1090 Vienna, Austria; 2 Department of Forensic Molecular Genetics, University Medicine Greifswald, Institute of Legal Medicine, 17489 Greifswald, Germany; 3 German Air Force Center of Aerospace Medicine, 82256 Fürstenfeldbruck, Germany; 4 Wallenberg Research Centre, Stellenbosch Institute for Advanced Study, 10 Marais Street, Stellenbosch, 7600 South Africa; 5 Present address: Institut für Neurobiologie, Universität Ulm, 89069 Ulm, Germany

**Keywords:** Neuroanatomy, Histology, Backfill experiments, Glomeruli, Chelicerata

## Abstract

**Background:**

Arachnids possess highly specialized and unorthodox sense organs, such as the unique pectines of Scorpiones and the malleoli of Solifugae. While the external morphology, numbers, and shapes of sensory organs are widely used in taxonomic studies, little is known about the internal anatomy of these organs and their associated processing neuropils in the central nervous system. Camel spiders (Solifugae) possess pedipalps and first walking legs heavily endowed with sensory structures, as well as conspicuous malleoli located ventrally on the proximal fourth walking legs. Malleoli are fan-shaped organs that contain tens of thousands of presumptive chemoreceptor neurons, but mechanoreceptive structures are absent.

**Results:**

Here, we examine the organization of the synganglion based on microCT analysis, 3D reconstruction of serial paraffin sections, and backfill preparations to trace the malleolar pathway. The projection area of malleolar afferents is intriguingly located in the most anterior ventral nerve cord, located in between the pedipalpal neuromere hemispheres. However, malleolar axon bundles are separated by a thin soma layer that points to an anteriad projection of the fourth walking leg neuromere. A conspicuous projection neuron tract that may receive additional input from pedipalpal sensory organs connects the malleolar neuropil with the mushroom bodies in the protocerebrum.

**Conclusion:**

Arthropod chemosensory appendages or organs and primary processing neuropils are typically located in the same segment, which also holds true in Solifugae, although the malleolar neuropil is partially shifted towards the pedipalpal neuromere. A comparison of the malleoli in Solifugae and the pectines in Scorpiones, and of their primary processing neuropils, reveals certain similarities, while striking differences are also evident. Similarities include the ventral arrangement of peg-shaped sensory structures on the respective segmental appendage, exposing dense arrays of chemoreceptive sensilla, and projections to a primary processing neuropil with glomerular subdivision. Differences are, e.g., the lack of mechanoreceptive afferents and an associated processing neuropil.

**Electronic supplementary material:**

The online version of this article (10.1186/s40851-019-0137-z) contains supplementary material, which is available to authorized users.

## Background

The central nervous system of chelicerates is among the least investigated in the arthropods. Compared with the wealth of studies in hexapods and crustaceans, no comparative overview on the neuroanatomy in Chelicerata in general, and especially in Arachnida, is available (e.g. [1, 2]). Although several studies have examined general aspects of neuroanatomical features in Chelicerata (e.g. [2–11]), detailed analyses are mostly present for single species such as *Limulus polyphemus, Cupiennius salei,* and various Salticidae and Scorpiones (e.g. [1, 2, 9, 12–21]). The euchelicerate central nervous system (excluding Pycnogonida) is situated in the prosoma and characterized by a fused mass comprising several neuromeres (termed synganglion) that encloses the esophagus (e.g. [2, 9, 22]). The supraesophageal part is the fused brain, consisting of the proto-, deuto- (alias cheliceral neuromere), and tritocerebrum (alias pedipalpal neuromere). However, a clear posterior border of the brain is not discernible and parts of the tritocerebrum clearly are located behind the esophagus. The subesophageal part comprises the neuromeres that are associated with the walking legs [2, 4, 21–25]. Within the brain, the protocerebrum comprises the most prominent neuropils: first and second order visual neuropils, as well as the paired mushroom body and the unpaired arcuate body [4]. The neuromeres associated with chelicerae and pedipalps possess mostly unstructured neuropilar regions. Arachnids are known to possess highly specialized sense organs, such as the mechanosensory slit sense organs [24], specialized cuticular sensilla [26], and trichobothria [27–29]. In certain taxa, hallmarking tactile and chemoreceptive sensory organs evolved [30–32]. While the external morphology, numbers and shapes of sensory organs and sensilla are widely used in taxonomic studies, little is known about the internal anatomy of these organs nor about associated processing neuropils in the central nervous system (e.g. [24, 32, 33]). In general, primary afferents of sensory organs together with local interneurons form dense synaptic processing units that are located in the associated segmental neuromere in the central nervous system. In the mandibulate brain (Myriapoda, Crustacea, and Hexapoda), primary processing neuropils associated with the chemosensory (first) antennae are the so-called olfactory lobes (also termed deutocerebral chemosensory lobes) [22, 34–36]. Here, axons of chemosensory receptor cells terminate in so-called olfactory glomeruli, which are the processing subunits of the olfactory lobe. In principle, an olfactory glomerulus is a more or less spheroid synaptic complex that may be ensheathed by glia cells. Chelicerates do not possess antennae, but often possess highly specialized chemosensory appendages. Associated with sensory (walking) legs, first order processing neuropils have been described in the ventral nerve cord of chelicerates [37, 38]. Accordingly, processing neuropils for chemosensory input may be located in any neuromere that is associated with chemosensory appendages, as has been described for many arachnids (e.g. [37, 39–44]). For example, olfactory glomeruli occur in association with the pectines of scorpions [21, 38], the first walking legs in Acari [41] and Amblypygi [45], or the pedipalps in Solifugae [40].

Another prominent example is the highly specialized malleoli of Solifugae, located on their fourth pair of walking legs. The possession and organization of these fan-shaped sensory organs is highly conserved [39]. In general, five malleoli are located on the ventral coxa, trochanter, and femur [46]. Each malleolus is composed of a stalk and a fan. While the shape of the fan may differ between species, it is always equipped with a ventral sensory groove. Numerous outer dendritic segments innervate the fan and project towards the groove [39, 47]. Based on fine structural analyses, Brownell and Farley [39] estimated approximately 72,000 receptor cells in each malleolus, which appears to be indicative of a pronounced chemoreceptive function. In fact, solifuges have often been observed probing the substrate with their malleoli at regular intervals when walking, perhaps searching for chemical cues to detect food sources or mates [46]. Furthermore, Wharton [48] reported on the mate search behavior of male *Metasolpuga picta* that locate females beneath the soil surface. Based on his observations, he suggested that female solifuges release a pheromone that might be detected via the malleoli. This may also explain a sexual dimorphism in the size of malleoli, which are typically larger in males. Beyond these few descriptions, very little is known on the behavioral use, functionality, or neurobiological characteristics of the malleolar system. This study sets out to investigate the basic pathway of malleolar receptor cell axons, as well as neuroanatomical features of primary processing neuropils based on microCT analysis, histology, and backfilling experiments. Additionally, we compare our results to the intriguing pectine appendages of Scorpiones and to the general architecture of chemosensory pathways in arthropods.

## Materials and methods

### Studied species

The present study is based on analyses of five species from three solifuge families. Voucher specimens of all species but *Galeodes arabs* are deposited in the Zoological Museum of the University of Greifswald (ZIMG), Germany. Specimens of *Nothopuga* sp. and *Oltacola chacoensis* Roewer, 1934 (Ammotrechidae) were collected in Argentina. Specimens of *Galeodes turkestanus* Kraepelin, 1899 (Galeodidae) originate from Kazakhstan and specimens of *Gluvia dorsalis* (Latreille, 1817) (Daesiidae) were collected in Portugal. Specimens of *Galeodes arabs* C.L. Koch, 1842 (Galeodidae) were collected near Sfax, Tunisia.

### Histology

For light microscopic investigations, prosomata of the above-listed species (except *G. arabs*) were fixed according to the method of Duboscq-Brasil [49]. After dehydration in ethanol, specimens were transferred to tetrahydrofuran and finally embedded in paraffin. Embedded specimens were serially sectioned (4–10 μm) with the rotary microtome Leica RM 2125 RT. Sections were stained with Azan according to the protocol of Geidies [49], and investigated and digitized with a Nikon Eclipse TE3000 microscope equipped with a Nikon DXM1200 camera.

### Backfilling experiments

**Fig. 1:**
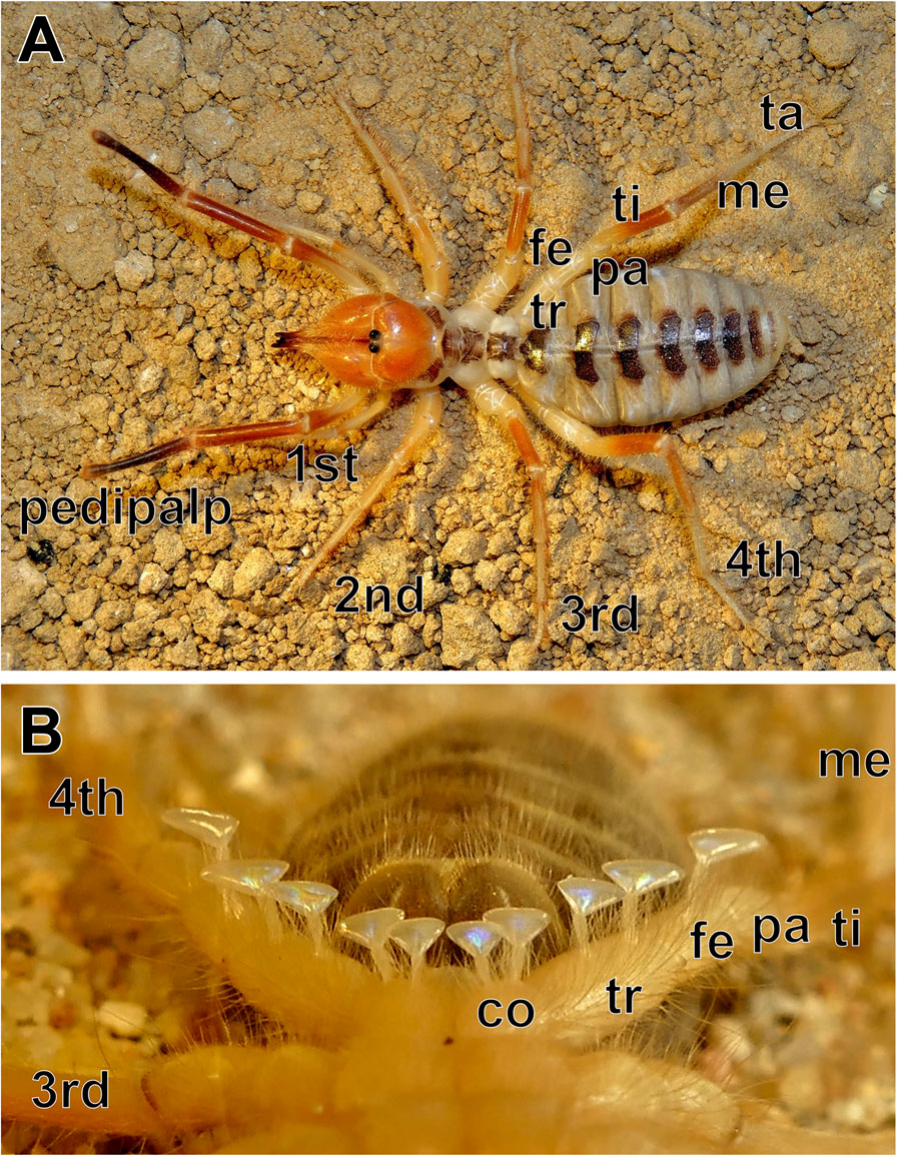
Camel spiders and their malleoli. **a** *Galeodes arabs*, dorsal view. **b** Anteroventral view of malleoli on the ventral surface of the fourth walking legs. Five malleoli are present on each leg, located on the coxa (or basis; 2 malleoli), trochanter (2 malleoli) and femur (1 malleolus). Abbreviations: co coxa, fe femur, me metatarsus, pa patella, ta tarsus, ti tibia, tr trochanter. Walking legs 1–4 are labelled 1st to 4th. Note that in **a**, the first walking legs are partly obscured by pedipalps

Several individuals of *Galeodes arabs* (Fig. 1a) were captured and processed within a few days, since solifuges usually do not survive in captivity for extended time periods. Standard backfill procedures [44, 50] were applied to nine specimens. Briefly, animals were cold-anesthetized by placing them into crushed ice for 15 min; all subsequent preparation procedures were carried out on a bed of crushed ice. Animals were pinned to a corkboard upside down to access the malleoli (Fig. 1b). A petroleum jelly well was erected around a selected malleolus, and the sensory organ was severed in its stalk. Distilled water was filled into the well for 20 min to widen the severed axons in the cut malleolus stalk osmotically. The water was replaced by a 1:1 mixture of NiCl_2_ and CoCl_2_ 0.5% solutions, and the animals placed in the refrigerator at 4 °C. The central nervous system was dissected after 32 to 48 h diffusion time, and rinsed in saline (20.47 g/l NaCl, 0.84 g/l KCl, 1.08 g/l CaCl_2_, 0.16 g/l MgCl_2_, buffered to pH 7.2 with TRIS [51]). Ni- and Co-ions were precipitated as rubeanic acid salts with dithiooxamide (one drop of saturated solution in 100% ethanol per 10 ml saline). The tissue was fixed (three parts saturated picric acid, one part 25% (v/v) glutaraldehyde, acetic acid added to 1%) overnight in the refrigerator, dehydrated in an ethanol series, and cleared in methyl salicylate. Detailed microscopic examination was conducted on three preparations with a Leica DM5500 B. Drawings for illustrations were made from merged image stacks on a graphic tablet (Huion 1060PLUS).

### X-ray micro-computed tomography (micro-CT)

Tomographic scans of two specimens of *G. dorsalis* were obtained using a Phoenix Nanotom 180 X-ray imaging system (Phoenix X-ray, part of GE Sensing & Inspection Technologies). Samples were fixed in Duboscq-Brasil, dehydrated in a graded series of ethanol and incubated in a 2% iodine solution (in pure ethanol) for 24 h [52]. After several washes in pure ethanol, samples were critically point dried (BAL-TEC CPD 030) and mounted on nail heads using cyanoacrylate glue. X-ray imaging was performed in high-resolution mode using the program datos|x acquisition (Target: Molybdenum, Mode: 0; Performance: 35 kV, 200 mA; Number of projections: 1000; Detector-Timing: 1500 ms; voxel size ca. 2 μm).

### 3D-visualization

Surface reconstructions were conducted based on microCT scans of *G. dorsalis*. Neuroanatomical features were reconstructed from serial sections of *G. turkestanus* and *Oltacola chacoensis* and from wholemount backfill preparations in *G. arabs*. Rough volume estimates of individual glomeruli in the *G. arabs* malleolar neuropil were performed in ImageJ [53]. Here, in each section, the area occupied by a given glomerulus was determined and multiplied by section thickness according to the microtome settings. For a given glomerulus, the volume portions from all sections containing that glomerulus were added up. Prior to surface reconstruction, serial sections of the respective prosomal synganglia of *G. tukestanus* and *O. chacoensis* were aligned elastically using the Fiji plugin TrakEM2 (according to [54]). Successive segmentation of the entire prosomal synganglia as well as major neuropils was conducted using Amira 5.6 (FEI, Visualization Science Group). Contours of structures of interest were delineated manually. The resulting label field was resampled (2;2;1) and a surface model generated using the “SurfaceGen” function. Further processing was performed using the “SmoothSurface” function and the surface editor.

For the creation of a 3D-PDF of the *O. chacoensis* nervous system (Additional file 1), Amira surfaces were exported as STL-files, processed in Fiji 3D viewer and imported to Adobe Acrobat Pro DC.

## Results

### General anatomy of the prosomal synganglion

**Fig. 2:**
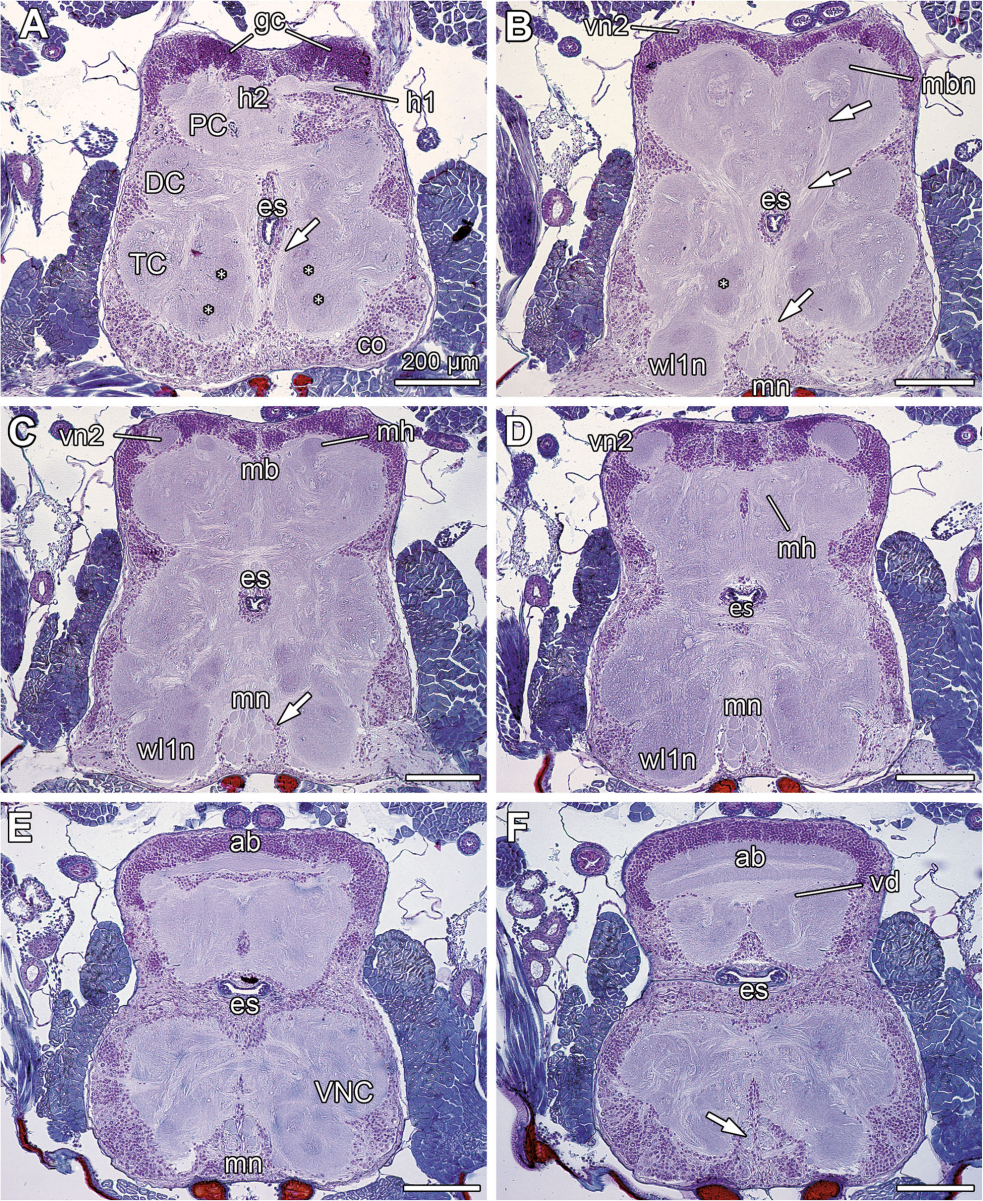
Selected transversal paraffin sections of the prosoma of *Galeodes turkestanus*, sorted from anterior to posterior. **a** Anterior synganglion with neuromeres of the proto- (PC), deuto- (DC), and tritocerebrum (TC). In the soma cortex, smaller and denser nuclei of the globuli cells supply the mushroom body. In the pedipalpal neuromere (TC), single glomerular neuropils are present (asterisks). **a**–**d** Two mushroom body hafts projecting posteriad are associated with the dorsally located mushroom body neuropil. Further posterior, both mushroom body hafts converge into the main haft. **a**, **b** The projection neuron tract (white arrows) projects dorsad to innervate the mushroom body neuropil. **b**–**d** The second order visual neuropil is embedded in the dorsal cortex. **b**–**e** The malleolar neuropil is composed of distinct glomerular neuropil parcels and ensheathed by a thin layer of somata (white arrow in C; see also Fig. 6a). **e**, **f** The arcuate body is located in the posterodorsal protocerebrum and subdivided into two lobes. **f** Paired axon bundles from the malleolar receptor neurons (neurite projection) are separated and ensheathed by soma cortex. The arcuate body is accompanied ventrally by a small, sickle-shaped domain (ventral domain). In this section, only the anterior parts are visible (further posterior the domain fuses medially; compare Fig. 3b, c). Abbreviations: ab arcuate body, co soma cortex, DC deutocerebrum (cheliceral ganglion), es esophagus, gc globuli cells, h1 mushroom body haft 1, h2 mushroom body haft 2, mb mushroom body, mbn mushroom body neuropil, mh mushroom body main haft, mn malleolar neuropil, PC protocerebrum, TC tritocerebrum (pedipalpal ganglion), vd ventral domain, vn2 second order visual neuropil, VNC ventral nerve cord, wl1n neuromere associated with the first walking leg. Scale bars = 200 μm

**Fig. 3:**
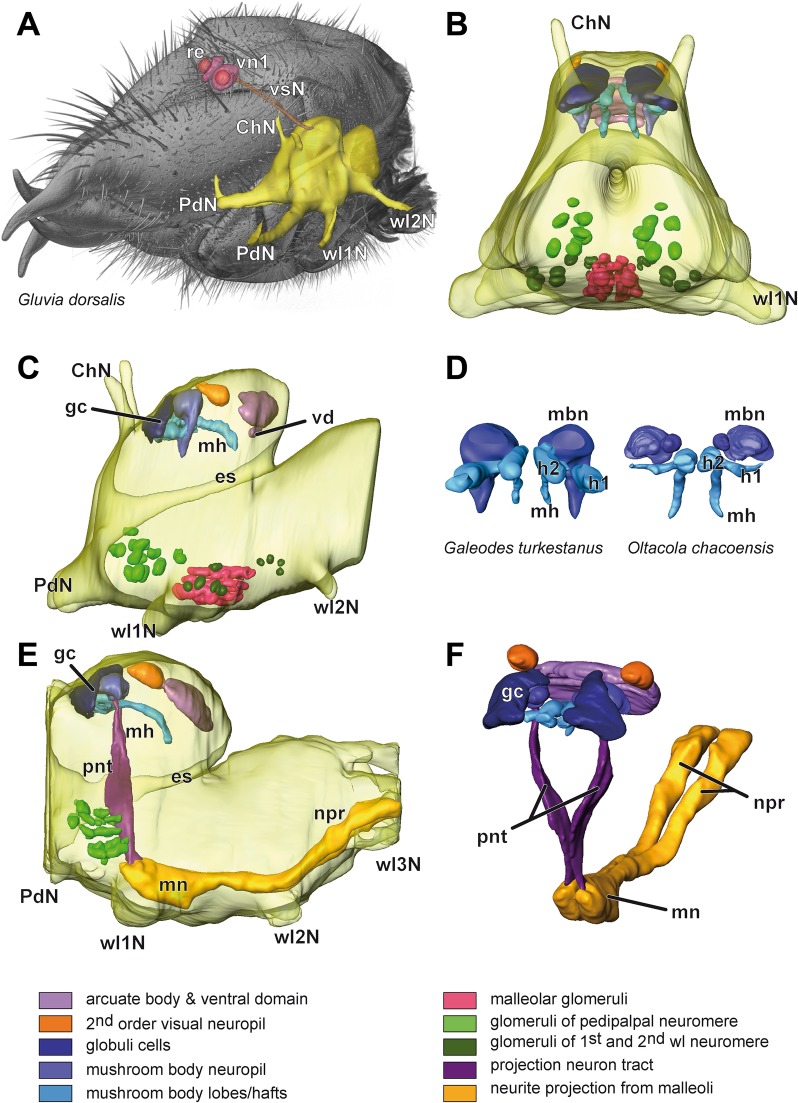
3D visualizations of the synganglia of *Gluvia dorsalis*, *Galeodes turkestanus* and *Oltacola chacoensis*. **a** Prosoma of *G. dorsalis* with anterior synganglion and parts of the visual system, based on microCT analysis. Note that the first order visual neuropils are closely associated with the retina of the primary eyes, and connected with the synganglion by long visual nerves (note: color of retina and lamina does not follow color code below). **b** Frontal view on the synganglion of *G. turkestanus*, based on paraffin sections. Color-coding of neuropils see below. The protocerebrum contains the major neuropils of the mushroom body and arcuate body. In the ventral synganglion, glomerular neuropils are evident associated with the malleoli (magenta), pedipalps (light green), and walking legs (darker green). **c** Lateral view on the synganglion of *G. turkestanus* (compare **b**). The arcuate body is located in the posteriormost protocerebrum; it is accompanied ventrally by a smaller ventral domain. The second order visual neuropil is located between arcuate body and mushroom body. The mushroom body consists of an anteriorly located globuli cell cluster, a mushroom body neuropil, and the two posteriad projecting hafts. Further posteriorly, the two hafts converge to the main haft. The malleolar neuropil is located at the level of the neuromeres of the first walking legs. Note the different volumes of glomerular neuropils associated with pedipalps and walking legs. Neurite projections and projection neuron tract omitted (compare **e**). **d** Reconstructions of the mushroom bodies of *G. turkestanus* and *O. chacoensis* (globuli cells omitted). In both species, two mushroom body hafts project posteriad and converge into the main haft. Note that in *O. chacoensis*, two mushroom body neuropils per hemisphere are present. **e** Lateral view on the synganglion of *O. chacoensis*, based on paraffin histology. General features are similar to *G. turkestanus* (compare **c**). Projections of the fourth walking leg neuromere (associated with the malleoli) proceed anteriad (darker yellow) and shape the malleolar neuropil. From the malleolar neuropil, a paired projection neuron tract (purple) proceeds dorsad to innervate the mushroom body neuropil. Anterodorsally, the malleolar neuropil is flanked by glomerular neuropils of the pedipalpal neuromere (green). See also interactive 3D visualization of this reconstruction in the Additional file 1. **f** Frontolateral view of the malleolar pathway and major neuropils in *O. chacoensis* (compare **e**). The projections with axonal elements of malleolar receptor cells proceed in distinct bundles. The projection neuron tracts approach each other, and then begin to diverge to provide space for the esophagus, diverging further dorsally (compare Fig. 4b). Abbreviations: h1, h2 mushroom body hafts, ChN nerve of chelicerae, es esophagus, gc globuli cells, mbn mushroom body neuropil, mh mushroom body main haft, mn malleolar neuropil, npr neurite projection of the fourth walking leg neuromere (associated with the malleoli), PdN nerve of pedipalpus, pnt projection neuron tract, re retina, vn1 first order visual neuropil, vd ventral domain, vsN visual nerve, wl1-3 N nerve of walking legs 1 to 3

**Fig. 4:**
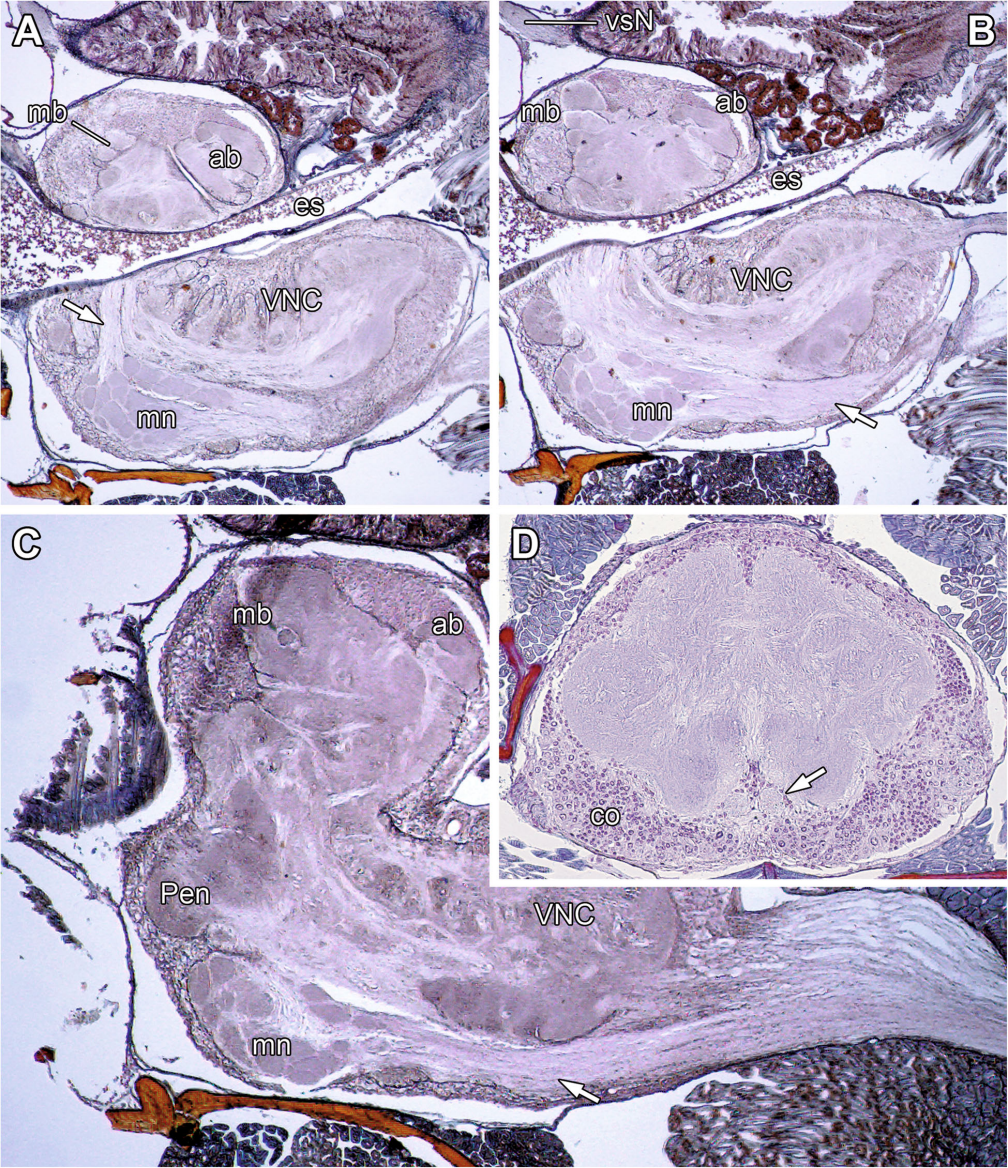
Selected sagittal and transversal paraffin sections of the synganglion of *Notophuga* sp. (**a**–**c**) and *Galeodes turkestanus* (**d**). **a**, **b** The arcuate body is located in the posterodorsal protocerebrum. Neuromeres associated with walking legs are separated from the malleolar projections by a thin cortex of somata (compare also **d**). The malleolar neuropil is located medially in the anteroventral synganglion, close to the pedipalpal neuromere. Note the bundle of malleolar afferent axons (white arrow in **b**). The projection neuron tract penetrates the cortex (compare also Fig. 2b) of the malleolar neuropil and proceeds dorsad (white arrow in **a**). **c** Paramedial section. Note the separation of the ventral fourth walking leg neuromere projection (white arrow) and the dorsal ventral nerve cord. Anteriorly, individual malleolar glomeruli can be distinguished. **d** Horizontal section of the *G. turkestanus* synganglion (prosoma midsection). The ventral nerve cord is enwrapped by a contiguous soma cortex, the malleolar projections (white arrow) are ensheathed by a thin layer of somata, embedded in the VNC. Abbreviations: ab arcuate body, co soma cortex, es esophagus, mb mushroom body, mn malleolar neuropil, Pen pedipalpal neuromere, VNC ventral nerve cord, vsN visual nerve

**Fig. 5:**
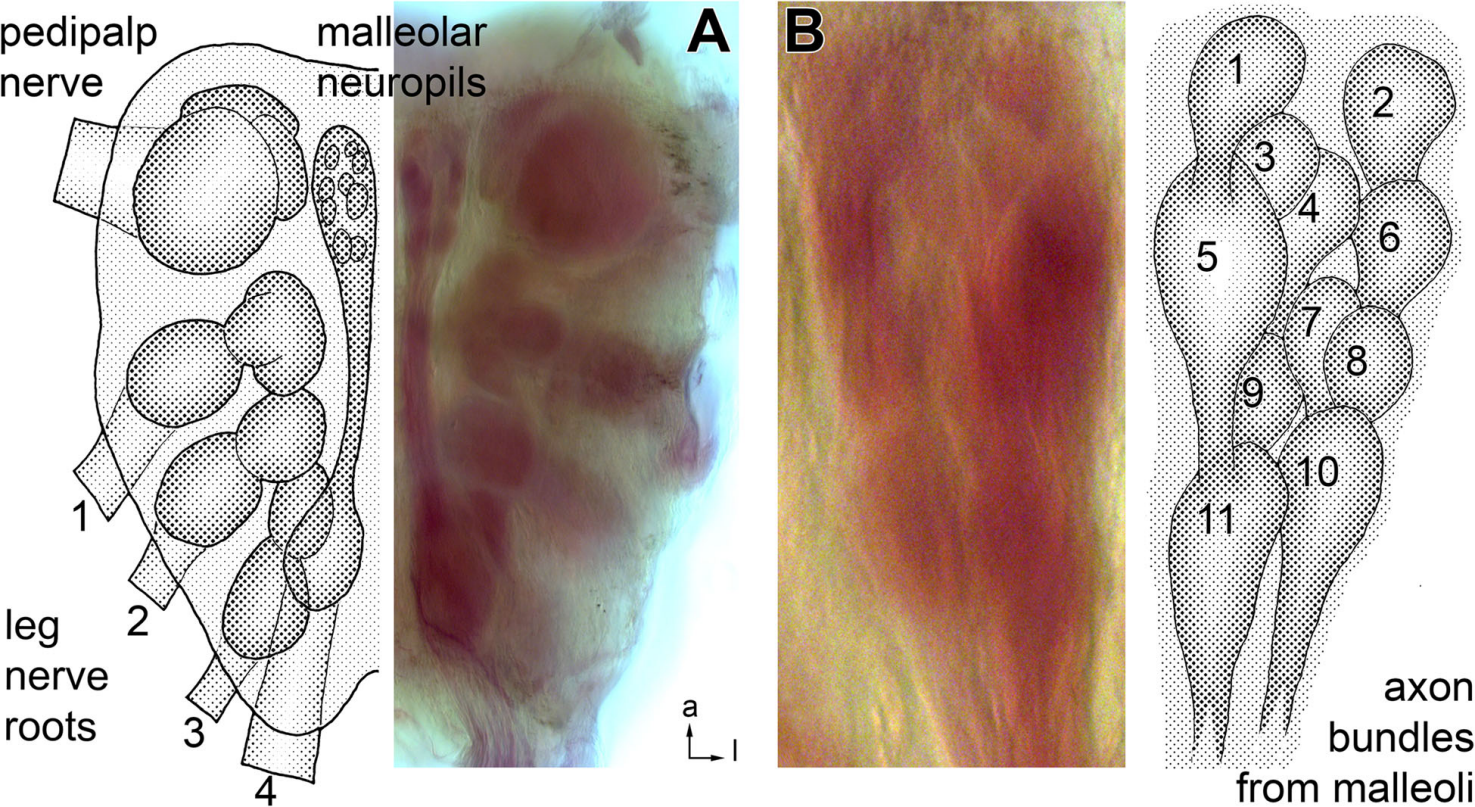
Glomerular organization of the malleolar neuropil, overview. **a** *Galeodes arabs* subesophageal synganglion in ventral view. Microscopic image of a malleolus backfill preparation (right) with corresponding schematic drawing (left). In this backfill, some Ni^++^-ions have seeped into all neuropils of the prosomal synganglion, providing an overview of ganglion anatomy. Nerve roots of pedipalp and first to fourth walking legs are labelled. The malleolar neuropils are located anteriorly close to the synganglion midline. **b** Detail from the preparation in A, taken from the other ganglion half. Drawing on the right outlines the 11–13 glomeruli that are identified when focusing through the depth of the preparation; glomeruli 5 and 11 may represent two separate glomeruli. Numbering is according to the appearance in transversal section series (Fig. 6b). Afferent axon supply from posterior is indicated. For all images, anterior is to the top and lateral to the right

The analyses from four different species showed similar results in nervous system architecture, as indicated by examples of histological sections and microCT analysis in *Galeodes turkestanus* (Figs. 2, 3b–d), *Oltacola chacoensis* (Fig. 3d–f; Additional file 1), and *Nothopuga* sp. (Fig. 4). The prosomal nervous system is a synganglion—a contiguous soma cortex ensheathes the fused neuromeres of the supraesophageal brain neuromeres and subesophageal neuromeres of the ventral nerve cord, distinct connectives are absent (Figs. 2, 3, 4). A clear separation of brain and ventral nerve cord is not evident (compare Figs. 2, 3, 4). The neuraxis of the brain is bent posterodorsally, resulting in a dorsal position of the protocerebrum (Figs. 2a; 3a–c, e; 4a–c). The protocerebrum comprises several major neuropils: the mushroom bodies, the second order visual neuropils of the primary eyes, and the arcuate body (Figs. 2, 3, 4). Each of the bilaterally symmetric mushroom bodies consists of a cup-shaped globuli cell cluster (Figs. 2a; 5c, e, f), several mushroom body lobes or hafts, and neuropilar domains (Figs.2a–d; 3b–f; 4a, b; Additional file 1) (terminology based on [2, 3, 9]). Two hafts project from the globuli cell cluster in mostly posterior direction (Figs. 2a–d, 3b–f). In *G. turkestanus* and *O. chacoensis*, an outer (haft 1; Fig. 3d) and an inner haft (haft 2; Fig. 3d) can be distinguished. Both hafts merge at the level of the mushroom body neuropils to the so-called main haft (Fig. 3b–e). A mushroom body bridge, linking both hemispheres, was not detected. Depending on the species, one or two mushroom body neuropils (glomerular domains) are present (Fig. 3b–f), which are innervated by the projection neuron tract (Fig. 3e, f; see also below). The cup-shaped first order visual neuropil of the primary eyes is located in close vicinity to the retina (Fig. 3a). The second order visual neuropil is located dorsally within the protocerebrum, between mushroom bodies and the arcuate body (Figs. 2c, d; 3b, c, e, f). Thus, first and second order visual neuropils are distinctly separated and connected by long neurites (Figs. 3a; 4b). The plan-convex arcuate body is located in the posterodorsal protocerebrum. Paraffin sections reveal two distinct layers (Fig. 2f). In *G. turkestanus*, the arcuate body is accompanied ventrally by a small, sickle-shaped domain (ventral domain; Figs. 2e; 3b, c).

The ventral nerve cord (subesophageal part of the synganglion) shows a conspicuous architecture. Ventrally to the neuromeres associated with the walking legs, paired medial projections from the malleoli extend anteriad (Figs. 2b–f; 3e, f; 4b, c; Additional file 1). The nerves of the fourth walking legs innervate the ventral nerve cord more laterally. The malleolar projections are separated from the neuromeres of walking leg ganglia by a thin, continuous cortex of somata (Figs. 2b–f, 4b–d). Thus, these projections are secondarily fused to and incorporated ventrally into the prosomal synganglion. The afferents proceed all the way to the malleolar neuropils, located in the most anteroventral part of the synganglion close to the midline (Figs. 2b–e; 3e, f; 4a–c; Additional file 1). The malleolar neuropil is composed of discrete glomeruli (see below). Finally, a paired projection neuron tract leaves the malleolar neuropil, penetrating the soma cortex that separates synganglion and malleolar projection, and proceeds dorsad through the pedipalpal and cheliceral neuromeres (Figs. 2a–c; 3e, f; 4a; Additional file 1). Ventral to the esophagus, the two projection neuron tracts approach each other, although crossing of neurites was not detected, and proceed dorsolaterad in direction to the neuropils of the mushroom body (Figs. 2b; 3e, f). Within the pedipalpal neuromere, 12–14 glomerular neuropil parcels are present per hemiganglion (Figs. 2a, b; 3b, c, e). Likewise, small glomerular neuropils are present in the neuromeres associated with the first and second walking legs (Fig. 3c).

### Malleoli and backfills of the malleolar axonal pathway

**Fig. 6:**
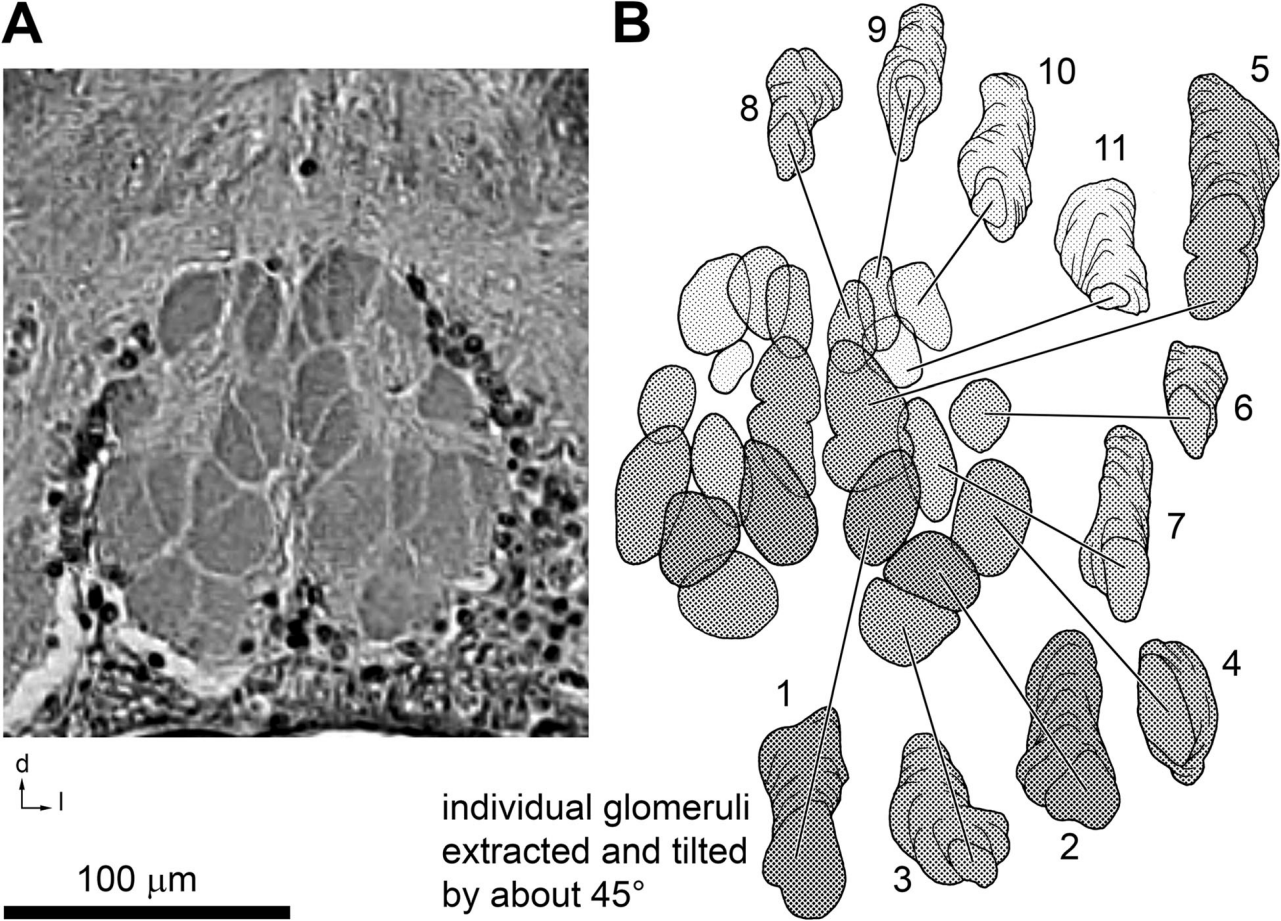
Glomerular organization of the malleolar neuropil, detail. **a** Transversal section through the anterior and ventralmost neuropil of the synganglion in *Galeodes turkestanus* (paraffin histology). Glomerular structures are clearly discernible in the compact malleolar neuropil, surrounded ventrally and laterally by somata, and dorsally and more laterally by neuropil of the pedipalpal neuromere. Ventrally, this histological section is delimited by the ganglion surface. **b** Schematic representation of individual glomeruli of the malleolar neuropil, frontal view. Glomeruli are shown as maximum projections along the longitudinal body axis, and thus partly appear to overlap due to their skewed arrangement (compare Figs. 3c and 4). More anterior and more posterior glomeruli are indicated in darker and lighter shadings, respectively. An exploded view of the glomerulus arrangement surrounds the neuropil reconstruction. Positions of the individual glomeruli are indicated by lines emerging from the frontal view. Numbering corresponds to Fig. 5b. The reconstructed glomeruli are tilted by about 45°, posterior end up, to illustrate their shapes and approximate sizes. Histological section planes (10 μm) are indicated by contour lines. For all images, dorsal is to the top and lateral to the right

Five malleoli are located on the ventral surface of each fourth walking leg. Coxa (also termed basis) and trochanter bear two malleoli each; the fifth malleolus resides on the femur (Fig. 1b). As stated above, malleolar afferents do not project to the proximal part of the segmental neuromeres of the fourth walking legs, but intriguingly to the distal part that is located in the anteriormost syncerebrum, the malleolar neuropils. Backfilling experiments revealed more specifically that a separate portion of axons associated with malleolar receptor neurons projects anteroventrally in two major neurite bundles. Backfills from one malleolus marked all glomeruli in one hemisphere of the synganglion, although with somewhat different intensities (Fig. 5a, b). Identical to histological analysis in *G. turkestanus, O. chacoensis* and *Notophuga* sp., in *G. arabs* the malleolar neuropils are located along the ventral midline in between the hemiganglia associated with the first walking legs and pedipalps (Figs. 3b, c, e; 5a). Axons in the neurite bundles strictly stay ipsilateral. Anteriorly, the neurite bundles widen and innervate the spheroid to ovoid malleolar glomeruli, with 10–12 glomeruli per hemisphere. Glomerulus length in the anterior-posterior axis is about two times the diameter of the rounded cross sections, rarely three to four times (Figs. 5b, 6). Glomerulus numbers depend on how strictly borders formed by axon bundles from the malleoli are interpreted as separating adjacent glomeruli (Figs. 5b; 6a; e.g. note tentative border within glomerulus no 5). The malleolar glomeruli are bilaterally almost, but not exactly, symmetric. Individual glomeruli may be of slightly different shape and location on the two body sides (Fig. 6). From wholemount backfill preparations in adult *G. arabs*, estimated volumes of glomeruli range from about 4000 to 24,000 μm^2^. The afferent axons innervate the malleolar neuropil area from posterior, supplying the glomeruli, and they proceed through the space between, and thus separate, neighboring glomeruli.

## Discussion

### General remarks on the nervous system of Solifugae

The nervous system of Solifugae was investigated briefly by Hanström [7, 55], Kästner [56] and Strausfeld [1], and in greater detail by Babu [3]. Our histological results and 3D reconstructions in general corroborate those of these earlier studies. Hanström [7] mostly referred to the wide separation of first (located outside the brain cortex) and second order visual neuropils of the primary eyes, which Kästner [56] highlighted to be a unique feature in arachnids. All authors described the architecture of the arcuate body subdivided into two lobes. Babu [3] also described an additional ventral domain (transverse commissural tract or neuropil sensu [3]; Fig. 3b, c) in close association with the arcuate body. In *Galeodes* sp. the possession of a third mushroom body haft was discussed by Hanström [55] and Babu [3]. However, we did not detect a third mushroom body haft ventrally to the main haft. According to Babu [3], glomerular masses associated with the mushroom body (mushroom body neuropils) are the target sites of the projection neuron tract arising from the malleolar neuropil (compare Figs. 2b, 3e, f; 4a). Babu [3] investigated several chelicerate representatives and stated that the paired projection neuron tract and a large sensory malleolar mass (malleolar neuropil) are unique to *Galeodes*. In addition, we detected glomerular neuropils in the pedipalpal neuromere and in the neuromeres associated with the first and second walking legs (Figs. 2a, b; 3b, c, e). In the pedipalpal neuromere, they were described as ‘pedipalpal nerve centers’ [3]. Strausfeld [1] stated that axons from chemoreceptors on the tips of the pedipalps innervate 14 glomeruli in the pedipalpal neuromere. Based on our 3D reconstruction, we confirm the presence of 12–14 glomerular neuropils in each pedipalpal hemiganglion (Fig. 3b, c, e; see below). However, for a more detailed investigation on the general neuroanatomy of Solifugae—especially with a focus on protocerebral neuropils—further immunohistochemical experiments are certainly desirable (e.g. [9, 57–59]).

### Sensory biology, axonal pathways, and malleolar neuropils

The anatomy of solifuge malleoli (or racquet-organs) has been investigated by Brownell and Farley [39] and Klann [47] and a pronounced chemoreceptive function was suggested. The 10 malleoli on the fourth legs form a linear sensory array spanning the body width. When the animal moves forward, the malleoli are often dragged passively over the substrate, and they probably also function in a near-field olfactory capacity ([27], and pers. observation AK, HW). A mechanoreceptive function can be excluded since tubular bodies were not detected [39]. However, the function of malleoli is still uncertain and further experiments, for instance, electrophysiological studies, are needed to prove their specific chemosensory function.

Axons of the malleolar receptor neurons project in two longitudinal bundles along the ventral nerve cord, separated from the surrounding neuromeres by a thin layer of somata (Figs. 2b–f; 3e, f; 4d; 5a). According to Babu [3], the paired axon bundles innervate the region between the hemispheres of the first walking leg neuromere, thus targeting a different neuromere. Based on our results we interpret the malleolar axons bundles ensheathed by somata as extensions of the neuromere associated with the fourth walking leg. They fuse secondarily with the anterior-most region of the ventral nerve cord between the pedipalpal neuromeres (Figs. 2c; 3e; 5a) (presumably during embryonic development). Generally, in arthropods, chemosensory appendages and their primary processing neuropils are located in the same segment (e.g. antenna-associated neuropils in the deutocerebrum). Strausfeld [1] argued that the situation in solifuges is an exception of this rule. However, based on our results we propose that the original view still holds true for Solifugae.

The malleolar neuropil is composed of distinct glomeruli. One of the first histological reports was given by Rühlemann [60] who hypothesized that malleoli are both chemo- and mechanoreceptive. Babu [3] termed this structure ‘malleolar sensory mass’. Strausfeld et al. [61] and Strausfeld and Reisenman [40] previously depicted the glomerular organization of the malleolar neuropil in Solifugae, but interpreted them to be associated (at least partially) with the pedipalpal neuromere. It is unclear whether these authors depicted both pedipalpal and malleolar glomerular neuropils (compare Fig. 7c, d in [40]). It is arguable that malleolar afferents terminating close to the pedipalpal neuromere may facilitate common processing of both mechano- and chemosensory input from sensilla associated with malleoli and pedipalps. After all, both structures are main sensory organs for probing the environment [56]. As mentioned above, the pedipalps as well as the first walking legs are tactile appendages, and the pedipalps have been proposed to have chemoreceptive function as well [62, 63]. As the projection neuron tract (which innervates the mushroom body) proceeds close to individual glomerular neuropils of the pedipalpal neuromere (Figs. 2b; 3e), the hypothesis of additional input to the projection neuron tract from pedipalpal sensory organs is conceivable. Strausfeld and Reisenman [40] mentioned that the projection neuron tract targets the mushroom body, which is corroborated by our histological data (Figs. 2b; 3e, f). These authors further focused on macroglomerular complexes in arthropods. Based on our backfill experiments and reconstruction of individual malleolar glomeruli, glomeruli volumes cover a broad range. Thus, the possession of macroglomeruli (in central and ventral positions; Fig. 6a, b) appears possible. However, it is not clear whether Strausfeld and Reisenman [40] differentiated small (pedipalpal) from large (malleolar) glomeruli. As we did not test for sex-specific differences, this might be an interesting topic for further investigation. Determining and differentiating volumes based on histological sections is always challenging. Shrinkage of sections in histology is notoriously difficult to assess, precluding proper volume estimates from reconstructed serial sections. The volume ranges for malleolar glomeruli of 4000–24,000 μm^2^ has thus to be considered with caution. It is the volume ratio of 1:6 that is interesting, more than the absolute numbers and volumes. Backfills from a single malleolus label all glomeruli in one hemisphere, albeit with different intensities. Ni^++^- and Co^++^- ions were used for the backfills, rendering this result a tentative one. Metal ions may pass into the interstitial space, particularly in the course of extended filling times, due to degenerating axons, thus ready to be taken up by adjacent fibers. If accurate, the present result would indicate functional organization of the malleolar neuropil comparable to glomerular subdivision in primary chemosensory neuropils of other animals, both invertebrates [22, 34, 35, 37, 64, 65] and vertebrates [66, 67].

## Conclusions

The pronounced glomerular organization of the malleolar neuropil is notable. It represents an important similarity to most primary chemosensory neuropils in animals (e.g. [68]; see above). This holds true despite differences in detail. Prominent is the afferent supply of the malleolar glomeruli from posterior, from the malleolar axons in which the glomeruli are embedded in. This innervation pattern differs from primary chemosensory processing neuropils in most arthropods that are supplied by afferent axons from almost all around their periphery, the afferents embracing the glomerular neuropil in a cage-like fashion before invading the neuropil, for example, in scorpions [44], in hexapods and in malacostracan crustaceans [34]. However, in centipedes, the array of chemosensory processing neuropils is innervated mostly unidirectionally [35, 69]. In some Chelicerata, sensory appendages are associated with characteristic and pronounced glomerular chemosensory processing neuropils in the ventral nerve cord, for example, in Scorpiones, Amblypygi, and Acari [1, 37, 41–44, 61]. The deutocerebrum of Mandibulata (innervated by the first antennae) is characterized by possession not only of a chemosensory, but also of a mechanosensory neuropil (apomorphic character complex, compare [35, 69]). Such a mechanosensory neuropil does not appear to be present in close association with glomerular neuropils in Chelicerata [1, 2, 35]. Brownell and Farley [39] only discussed a chemosensory function of malleoli, as typical tubular bodies for detecting mechanical stimuli were not described in ultrastructural studies. In general, primary processing of mechanosensory signals from arachnid walking legs occurs in longitudinal tracts and neuropils in the ventral nerve cord [4, 24, 27]. In any case, our backfill experiments did not detect any labelled neuropils other than the malleolar glomeruli.

A comparison of the malleoli in Solifugae and the pectines in Scorpiones, and of their primary processing neuropils, reveals intriguing similarities, but also clear differences. The list of similarities starts with the arrangement of the sensory structures—sensory pegs on scorpion pectines and malleoli on solifuge walking legs—in rows along the ventral surface of the segmental appendage. Scorpion pectines are chemo- and mechanosensory appendages. Both functions were proven by ultrastructural analyses [70] and electrophysiological experiments [15]. In addition to chemoreceptive dendrites, scorpion peg sensilla contain a single mechanoreceptive dendrite at the peg base characterized by a tubular body [70]. Axons from scorpion pectines project into two separate neuropils, a presumably chemosensory neuropil with a glomerular (or rather lobular) structure, and an unstructured, presumably mechanosensory neuropil [38, 44]. Such a separation into two neuropils is not evident in Solifugae and surely is due to the lack of mechanoreceptive elements in the malleoli. A peculiar similarity of primary processing neuropils in scorpions and solifuges is the anteriad projection of afferents and neuropils. However, based on the study by Wolf [44] it is not clear whether axonal projections and neuropils are ensheathed by a soma cortex as seen in solifuges. It is possible that the neuropils associated with the pectines are also an anteriad projection embedded in the ventral nerve cord that is secondarily fused to it (compare fig. 6c in [44]).

## Additional file

Additional file 1: Interactive 3D visualization of the nervous system of *Oltacola chacoensis* based on Amira reconstruction of paraffin sections (compare Fig. 3e, f). To activate, click on the figure in Adobe Reader and by using the computer mouse you can bring the model in any desired position and magnification. Using the model hierarchy, you can in- or exclude different components. Note that the left neurite projection was omitted to display the structure of the malleolar glomeruli. (PDF 16747 kb)

## Data Availability

The data generated and/or analyzed during the current study are available from the corresponding author upon reasonable request.
