# Supplementary material for: Primary processing neuropils associated with the malleoli of camel spiders (Arachnida, Solifugae): a re-evaluation of axonal pathways
Source: Zoological Lett. 2019 Aug 2;5:26. doi: 10.1186/s40851-019-0137-z (PMC6679463; doi:10.1186/s40851-019-0137-z)
Supplement: Supplementary file 1 — Interactive 3D visualization of the nervous system of Oltacola chacoensis based on Amira reconstruction of paraffin sections (compare Fig. 3e, f). To activate, click on the figure in Adobe Reader and by using the computer mouse you can bring the model in any desired position and magnification. Using the model hierarchy, you can in- or exclude different components. Note that the left neurite projection was omitted to display the structure of the malleolar glomeruli. (PDF 16747 kb) [file 40851_2019_137_MOESM1_ESM.pdf]

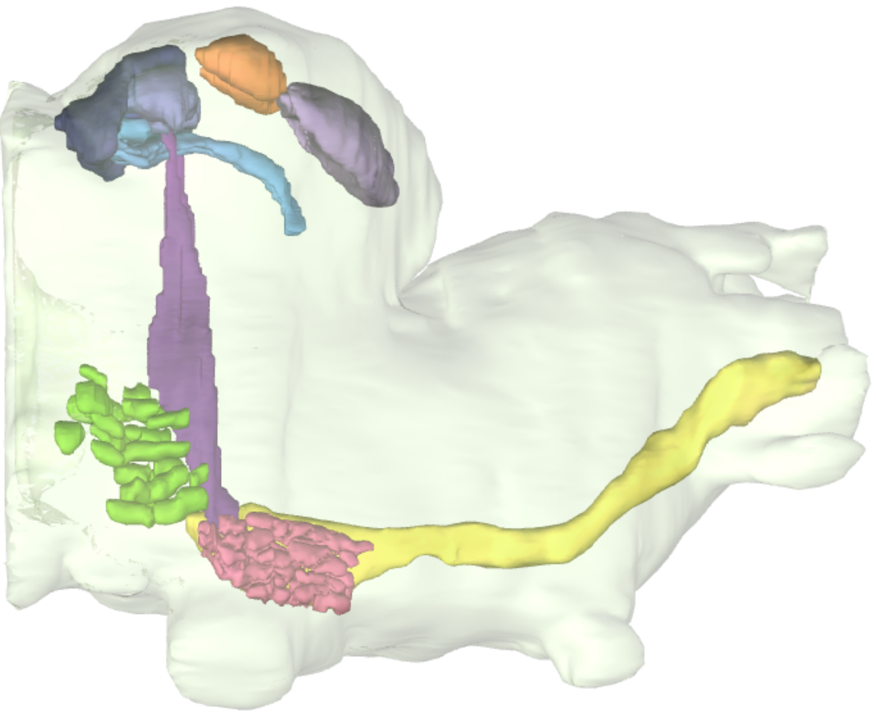

Interactive 3D visualization of the nervous system of *Oltacola chacoensis* based on Amira reconstruction of paraffin sections (compare Figure 3E, F). To activate, click on the figure in Adobe Reader and by using the computer mouse you can bring the model in any desired position and magnification. Using the model hierarchy, you can in- or exclude different components.
Note that the left neurite projection was omitted to display the structure of the malleolar glomeruli.
